# A comparative study of subjective well-being, interpersonal relationship and love forgiveness among Chinese college students before and after the COVID-19 epidemic

**DOI:** 10.3389/fpsyg.2023.1229451

**Published:** 2023-07-28

**Authors:** Tianyi Cheng, Lin Qiyi, Hong Fu

**Affiliations:** ^1^College of Educational Sciences, Huaiyin Normal University, Huaian, Jiangsu, China; ^2^College of Psychology, Nanjing Normal University, Nanjing, Jiangsu, China

**Keywords:** Chinese college students, the COVID-19 pandemic, interpersonal relationships, subjective well-being, love forgiveness

## Abstract

During the period that COVID-19 pandemic outbreak, Chinese universities have adopted a new teaching method combining online and offline and banned students from entering and leaving campus at will in line with the epidemic prevention policy. As a result, college students’ learning and life styles have been greatly changed. In order to explore how the epidemic and specific prevention policies have influenced the psychology and behavior of Chinese college students, this study used three questionnaires of college students’ subjective well-being, interpersonal relationship and love forgiveness to collect the data after the epidemic and compared with the data of college students before the epidemic. The result showed that before and after the epidemic, college students had obvious changes in their level of interpersonal relationship, subjective well-being and love forgiveness. Relationships among the three variables had changed. Meanwhile, the demographic variables of college students had certain changes in the three questionnaires. College students with and without romantic experience also had significant differences in the three questionnaires. It can be seen that the epidemic and specific prevention policies have a certain impact on the physical and mental health of college students, and there is also a gap in the forgiveness level of college students with and without romantic experience. These findings remind relevant departments that it should give greater consideration to the physical and mental health of college students, provide some references for dealing with new outbreaks and formulating the epidemic prevention policies subsequently, and suggest psychological counselors to change the way of dealing with the intimate relationship of college students.

## Introduction

The outbreak of COVID-19 pandemic has had diverse impacts on people’s study, work and even life, and dealt a heavy blow to the physical and mental well-being of countless individuals (Talevi et al., 2020; Chen et al., 2021; Xie et al., 2022). The study of de Abreu et al. (2021) found that adolescents’ fear of possible infection with the virus added their anxiety, which would lead to more serious emotional problems (Xie et al., 2020).

On 5 December 2022, China made an official announcement on the conclusion of the COVID-19 outbreak. During the epidemic, China had taken special epidemic prevention measures for 3 years. Chinese universities adopted a new teaching method combining online and offline, and closed campus gates and barred students from entering and leaving the campus as they please to reduce the possibility of the virus spreading at the universities. On the one hand, epidemic prevention measures have disrupted the existing educational form, mode and learning style of Chinese higher education (Sun et al., 2022), and also changed the lifestyle of students. In school, students not only face the uncertainty of when the pandemic will end, but also have to adjust to new learning methods (Eva et al., 2021). Engaging in online education reduces the level of interaction between students and teachers, resulting in heightened learning fatigue and provoking negative emotions like stress (Muslim, 2020). On the other hand, epidemic prevention measures may do harm to students’ mental health. Evidences showed that the measures affect people’s mental health in a negative way (Chew et al., 2020; Qiu et al., 2020; Rossi et al., 2020; Wang et al., 2020). Additionally, Krautter et al. (2022) found that college students were significantly affected by various restrictions imposed during the epidemic.

Yaremtchuk and Bakina (2021) argue that adolescents are among the most vulnerable groups and it is important to study their response during an epidemic. Studies have shown that the COVID-19 pandemic has influenced college students’ mental well-being and behavioral pattern (Son et al., 2020; Gestsdottir et al., 2021; Li et al., 2021; Gadi et al., 2022; Zhu and Shen, 2022; Patin et al., 2023; Wu, 2023). Individuals’ subjective well-being shows their life satisfaction, positive and negative emotional experience as well (Diener et al., 1999), which holds great importance in individuals’ mental health (Steinmayr et al., 2019). Subjective well-being of college students can serve as an indirect indicator of their mental health (Eva et al., 2021). According to Zhang F. et al. (2021), undergraduates’ subjective well-being in college during the epidemic period was at an above average level, which is consistent with the study results before the epidemic period. However, the study of Baetens et al. (2022) shows the pandemic does adversely affect undergraduates’ subjective well-being in college. Chen and Ye (2023) found that compared with the period before the epidemic, undergraduates’ subjective well-being in college had decreased significantly during the COVID-19. Krautter et al. (2022) found that during the epidemic, the subjective well-being of college students decreased.

College students’ subjective well-being is associated with interpersonal relationship (Jiang and Tan, 2016; Cheng et al., 2021). Interpersonal relationship refers to the mode of contact and interaction between people (Ma, 2022). College students’ interpersonal relationship is a kind of external expression of their behavior, revealing their behavioral patter. The epidemic has changed people’s interpersonal relationships (Tribowo et al., 2021) and has multiple effects on interpersonal relationships in school (Herrmann et al., 2021). Zhang X. et al. (2021) investigated the relationships among Chinese college students’ interpersonal relationships, school adjustment, mental resilience and social support in the period of pandemic. This study emphasized the role of interpersonal relationship and found a strong association between interpersonal relationship and school adjustment. Nevertheless, the study did not discuss the changes in college students’ interpersonal relationships before and after the pandemic. At present, there is a lack of research on the changes of interpersonal relationship among undergraduates before and after the epidemic.

A romantic relationship is a special kind of human relationship. Love forgiveness refers to the forgiveness within a specific relationship and it pertains to an individual’s inclination to forgive for his specific object of interpersonal relationship (Paleari et al., 2009; Zhang and Fu, 2014). This forgiving tendency includes emotion, cognition and behavior (Enright et al., 1992), involving both individual psychology and behavior. The level of love forgiveness of undergraduates can reflect their psychological health and behavioral pattern indirectly. The studies showed that interpersonal forgiveness is beneficial to individuals’ mental health (Berry et al., 2005; Chen et al., 2017). Therefore, it is of certain significance to investigate the love forgiveness of undergraduates after the epidemic. Tribowo et al. (2021) investigated the relationships of married people in Indonesia and pointed out that the pandemic has changed the relationships of couples. Overall et al. (2021) also pointed out that the pandemic has a serious impact on couples’ relationships. However, there is no evidence that the pandemic has changed relationships among college students. In addition, Love forgiveness in college students is correlated with subjective well-being (Cheng et al., 2021). Study has shown that individual’s forgiveness is negatively correlated with negative emotional experience during the pandemic (Tilkeridou et al., 2021). The epidemic can increase individual’s negative emotions (Genc and Arslan, 2021). Therefore, the epidemic may change the level of love forgiveness among university students. However, as far as we know, no researchers have investigated the undergraduates’ love forgiveness after the pandemic.

Existing researches about the influence of the pandemic upon undergraduates’ subjective well-being have shown inconsistent results. This problem needs more discussion. Meanwhile, to our knowledge, it is unclear whether college students’ interpersonal relationships and love forgiveness have changed during the pandemic. Until now, no researchers have compared the three variables among Chinese university students before and after the pandemic period. A study comparing the three variables among Chinese university students before and after the time of the COVID-19 pandemic could be helpful for us to understand whether the COVID-19 pandemic have influenced Chinese university students and Chinese special epidemic prevention policies from both psychological and behavioral aspects. Firstly, this can provide some evidence from Chinese college students for relevant researches about the effects of COVID-19 epidemic and prevention policies. Secondly, this research is helpful for other researchers to know the current situation of students’ well-being, interpersonal relationships and intimate relationships. Thirdly, the results of the study can provide some references for relevant departments to formulate policies conducive to individuals’ mental health when dealing with the epidemic in the future.

The data collected before the epidemic were all from college students with romantic experience. To enhance the ecological validity of the research conclusions, this research tried to compare the differences in interpersonal relationship, subjective well-being and love forgiveness of undergraduates with and without romantic experience. The love forgiveness among university students without romantic experience is their imagined forgiveness. Comparing the love forgiveness between university students with and without romantic experience can help researchers figure out the distinction between the imagined and the actual love forgiveness. At present, the research in this area is still relatively lacking. This article can not only enrich the research results in the field of intimate relationship and forgiveness, but also provide more possible ideas for improving individuals’ mental health.

Overall, this article aims to explore the impact of COVID-19 and epidemic prevention policies in China on college students by comparing the subjective well-being, interpersonal relationship and love forgiveness before and after the epidemic. In addition, this study also explored the differences in the three variables among undergraduates with and without romantic experience. Therefore, our attention is directed toward these unexplored questions:

*Research Question 1*: Do Chinese college students’ love forgiveness, subjective well-being and interpersonal relationships change before and after the time of pandemic?

*Research Question 2*: Have the three variables of Chinese college students of different genders changed before and after the pandemic?

*Research Question 3*: Have the three variables of Chinese college students of different grades changed before and after the pandemic?

*Research Question 4*: Have the three variables of Chinese college students of different regions changed before and after the epidemic?

*Research Question 5*: Are there differences in three variables between college students with romantic experience and those without romantic experience?

Based on questions of the research, we put forward the related hypotheses below:

*Hypothesis 1*: The subjective well-being, interpersonal relationship and love forgiveness among Chinese undergraduates will change significantly before and after the epidemic.

*Hypothesis 2*: Chinese college students of different genders will show significant changes in the three variables before and after the epidemic.

*Hypothesis 3*: Chinese college students of different grades will show significant changes in the three variables before and after the epidemic.

*Hypothesis 4*: Chinese college students of different regions will show significant changes in the three variables before and after the epidemic.

*Hypothesis 5*: There are significant differences between college students with romantic experience and those without romantic experience in three variables.

## Materials and methods

### Participants

Before the pandemic, we surveyed a group of Chinese college students who had a romantic relationship in 2016. After the pandemic, we surveyed a group of Chinese college students in 2023, who were divided into two categories: those who had a romantic relationship and those who did not. Due to the 7-year interval between the two surveys and the anonymous data collection method we used, the subjects of the two surveys were not the same group of college students, which should be emphasized.

In 2016, Chinese university students who have romantic experience were invited take part in the survey. We choose the subjects at random and distributed paper questionnaires in university across China, as well as online. The paper questionnaires were 761 and online questionnaires were 179. After excluding 109 invalid questionnaires, a total of 831 valid questionnaires were got; 412 questionnaires were from male and 419 from female; 286 data were from freshmen, 159 were from sophomores, 192 were from juniors, and 194 were from seniors. The data from urban areas and rural areas were, respectively, 468 and 363.

In 2023, Chinese college students were invited take part in the survey. We choose the subjects at random and the subjects were asked to fill out the questionnaires online. After excluding 160 invalid questionnaires, a total of 1,641 valid questionnaires were got; 976 students with romantic experience and 505 students without romantic experience. In terms of gender, the subjects included 601 males and 880 females. Regarding grade level, there were 423 freshmen, 444 sophomores, 315 juniors, and 299 seniors. Concerning geography, the data from urban areas and rural areas were, respectively, 697 and 784.

### Measures

The study adopt three scales based on Chinese university students to explore the changes of Chinese undergraduates’ subjective well-being, interpersonal relationship and love forgiveness before and after the COVID-19 epidemic, as well as the difference among undergraduates with and without romantic experience: the College Students’ Love Forgiveness Questionnaire, College Students’ Interpersonal Relationship Comprehensive Diagnostic Scale, and College Students’ Subjective Well-being Questionnaire. For convenience, the three scales are abbreviated as CS-LFQ, CS-IRCDS, and CS-SWQ.

### College students’ subjective well-being questionnaire

Jiang and Yang (2008) compiled the CS-SWQ. The questionnaire is divided into 8 factors and includes 61 questions and some reverse questions. The score of the CS-SWQ is comprised of five levels, ranging from 1 which denotes complete inconsistency to 5 which represents complete consistency. The score is higher, the level of subjective well-being is higher. The CS-SWQ’s Cronbach alpha coefficient is 0.959, and the alpha coefficient of each factor falls within the range from 0.765 to 0.916. The CS-SWQ had good content validity, construct validity and calibration validity. The questionnaire was employed by Jiang and Bai (2009) and Jiang and Tan (2016), both of which got positive outcomes.

### College students’ interpersonal relationship comprehensive diagnostic scale

The CS-IRCDS was compiled by Zheng et al. (2005), which is divided into 4 dimensions. The questionnaire includes 28 questions, which has some reverse questions. The score for each question is either 0 or 1 (with 0 being non-conformity, 1 being conformity). Individuals with higher total scores tend to have better relationships. The cumulative score obtained from the questionnaire between 0–8, 9–14, and 15–28, respectively, indicates trouble getting along with friends, somewhat difficult to maintain relationships with friends, and minimal interpersonal troubles. The reliability indexes of each subscale of the questionnaire and the validity were all good. The scale’s total score exhibited a high level of internal consistency with an alpha coefficient of 0.82. The questionnaire was employed by Cui et al. (2015) and Jiang and Tan (2016), which had good results.

### College students’ love forgiveness questionnaire

Zhang and Fu (2014) compiled the CS-LFQ, and set 27 questions. The questionnaire contain four dimensions, which are revenge, avoidance, forgiveness, and negative contemplation. The score of questionnaire is six levels, ranging from 1 which denotes complete consistency to 6 which represents complete inconsistency. The revenge, avoidance and negative contemplation are negative dimensions. The forgiveness is positive dimension and has reverse questions. After some questions are scored in reverse, a higher total score indicates a greater degree of forgiveness. The four dimensions of the scale demonstrated good internal consistency with alpha coefficients of 0.735, 0.862, 0.877, and 0.892, respectively. The total questionnaire showed excellent internal consistency with an alpha coefficient of 0.897, and exhibited strong structural and external validity. The CS-LFQ has been used by the studies of Liu (2017), Sun et al. (2018), and Yu (2019), which had good results.

### Procedure

**Figure 1:**
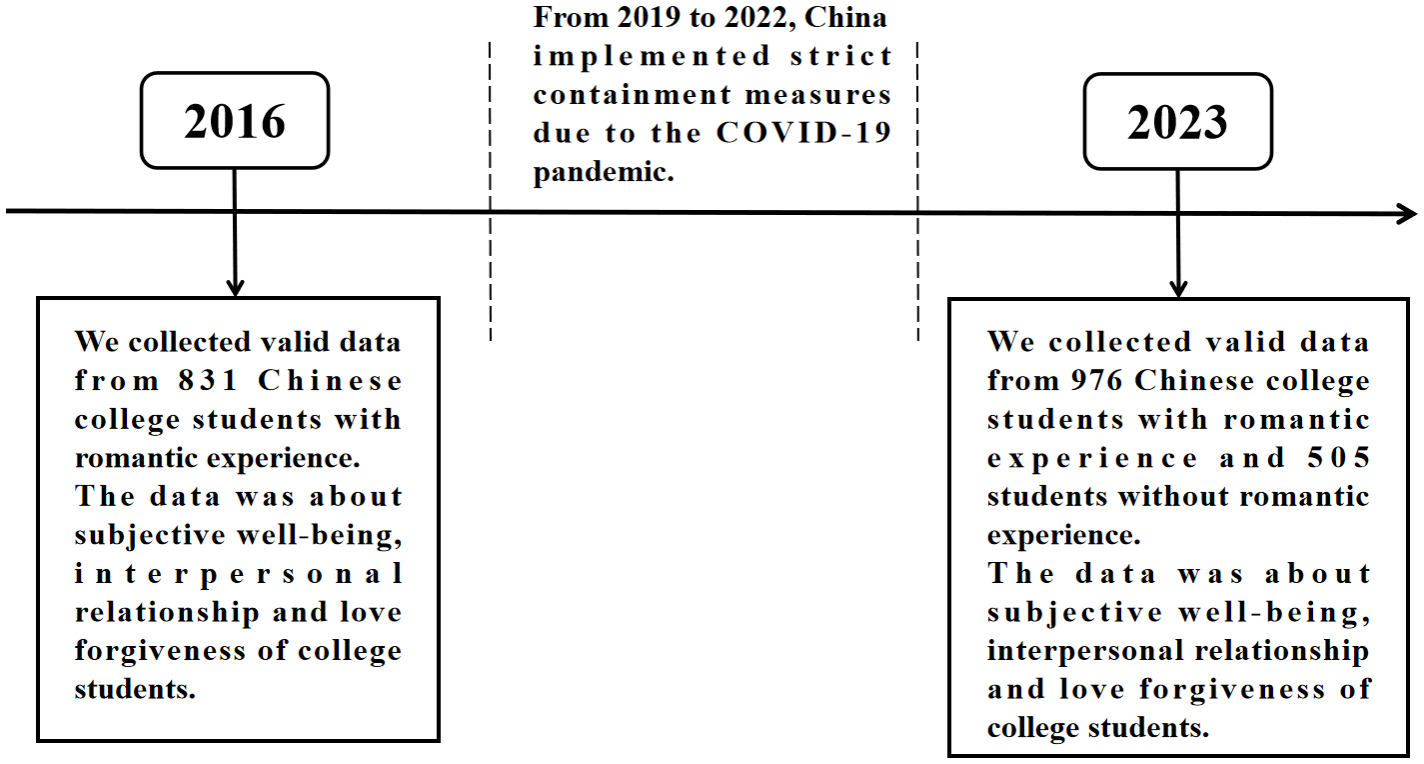
Experimental procedure flow chart.

Before the pandemic, we surveyed a group of Chinese college students who had a romantic relationship in 2016. After the pandemic, we surveyed a group of Chinese college students in 2023, who were divided into two categories: those who had a romantic relationship and those who did not. Due to the 7-year interval between the two surveys and the anonymous data collection method we used, the subjects of the two surveys were not the same group of college students, which should be emphasized. The flow chart of the experimental procedure is shown in Figure 1.

In 2016, we bound the three questionnaires into a book and invited college students to respond at random. Initially, the participants were asked whether they had romantic relationships and their willingness to participate in the research. If the students had previous romantic involvement or were currently in a romantic relationship, and expressed their willingness to participate, they were subsequently requested to complete the scales. The survey process lasted for about 20 min and was carried out anonymously. Once the questionnaires were all gathered, it underwent a verification and screening process, and data from the valid questionnaires were entered.

In 2023, we combined the three questionnaires into one online questionnaire and invited college students to respond during their rest. Only after obtaining the consent of the subjects, we would ask them to complete the online questionnaire. If college students had no romantic relationship, according to the instructions in the questionnaire, they should complete the selection of relevant content with imaginary. The anonymous online survey of approximately 20 min in duration was carried out. Once questionnaires were all gathered, we checked and screened all the questionnaires.

Approval for this study was granted by the ethics committee at the School of Psychology, Nanjing Normal University and the ethics committee at the School of Educational Sciences, Huaiyin Normal University. We informed all subjects of the main purpose of the study and obtained their consent.

### Statistical analysis of data

The data obtained in this study were statistically analyzed by SPSS27.0 and Mplus8.3 software, and the statistical methods used are mainly descriptive statistics: *t*-test, *F*-test, and a structural variance model.

## Results

### Descriptive statistics

#### Reliability analysis

In both 2016 and 2023, the internal consistency coefficients of the three questionnaires were all above 0.80. Therefore, the data were deemed to be reliable. We conducted a common method bias test on all the data from 2016 and 2023 by using SPSS27.0. The Harman’s single factor test results showed that the first unrotated factor explained only 16.79% of the total variance, accounting for 40% of the total explanatory variance. Therefore, we believe that there is no severe common method bias present.

#### Difference analysis

The average scores and standard deviations of participants with romantic experience in three scales were presented in Table 1 for both the years 2016 and 2023. Multivariate analysis of variance was performed for the CS-LFQ, demographic variables and years. However, the result of homogeneity test of variance was significant (*F* = 9.492, *p* < 0.001), indicating that conducting this analysis would not be appropriate. By examination, a multivariate analysis of variance was also unsuitable for CS-IRCDS and CS-SWQ. Therefore, we conducted *t* tests on the data in 2016 and 2023. The data from 2 years were analyzed by using independent sample t-test through SPSS27.0. According to the results, there was a significant difference between the 2 years in terms of CS-SWQ (*t* = 19.532, *p* < 0.001, *Cohen’s d* = 0.919), CS-IRCDS (*t* = −0.646, *p* < 0.001, *Cohen’s d* = 0.031), and CS-LFQ (*t* = 8.674, *p* = 0.005, *Cohen’s d* = 0.408). The small *Cohen’s d* values in the CS-IRCDS need to be given special attention. College students’ average scores in the CS-IRCDS of 2 years were very close, but the standard deviation was not. The average scores became lower in both CS-SWQ and CS-LFQ in 2023. Therefore, it can be concluded that undergraduates’ subjective well-being, love forgiveness, and interpersonal relationships changed significantly before and after the epidemic.

**Table 1:** Mean and standard deviation of three questionnaires in 2016 and 2023.

	College students	SW	IRCDS	LF
2016	With romantic experience	221.079 ± 36.8547	19.110 ± 5.200	73.096 ± 14.552
2023	With romantic experience	193.105 ± 23.422	19.288 ± 6.345	66.809 ± 16.007
	Without romantic experience	187.812 ± 22.746	18.032 ± 6.615	68.633 ± 17.695

Table 2 presents the mean scores and standard deviations of students in the CS-SWQ across different dimensions in 2016 and 2023. We attempted to perform a one-way ANOVA on different dimensions across different years. However, the results of the homogeneity of variance test showed significance. Therefore, we conducted independent samples *t* tests for each dimension separately, and the results are presented in Table 2. Except for the dimension of positive emotions, the other 7 dimensions showed significant differences across different years. Due to reverse scoring, lower scores on negative emotions indicate more negative emotions. Comparing to 2016, college students in 2023 have more negative emotions and improved romantic relationships. However, there has been a decline in five other dimensions: evaluation of academic and life, sense of career and employment, self-evaluation, interpersonal communication and connection, and academic achievement and experience.

**Table 2:** Mean, standard deviation, and *t*-test in different dimensions of CS-SWQ.

	2016	2023	*t*	*p*	*Cohen’s d*
Negative emotion	54.490 ± 10.521	38.554 ± 12.215	29.441^**^	<0.001	11.467
Positive emotion	41.697 ± 7.903	41.310 ± 7.587	1.058	0.290	7.736
Evaluation of academic and life	32.460 ± 6.107	30.131 ± 4.220	9.535^**^	<0.001	5.174
Sense of career and employment	20.588 ± 4.711	18.598 ± 3.561	10.209^**^	<0.001	4.130
Romantic relationships and emotions	16.631 ± 5.294	18.63 ± 4.6597	−8.569^**^	<0.001	4.961
Self-evaluation	14.284 ± 3.102	11.809 ± 2.729	18.038^**^	<0.001	2.906
Interpersonal communication and connection	19.184 ± 3.704	16.778 ± 2.471	16.447^**^	<0.001	3.100
Academic achievement and experience	21.746 ± 4.135	17.287 ± 2.996	26.499^**^	<0.001	3.565

^**^p < 0.01.

Table 3 displays the mean scores and standard deviations of students in the CS-IRCDS across different dimensions in 2016 and 2023. The results of independent samples *t* tests for each dimension are shown in Table 3. College students only show significant differences in the two dimensions of interacting with others and interacting with the opposite sex. College students in 2023 scored higher on the dimension of interacting with others and lower on the dimension of interacting with the opposite sex. It should be noted that in the CS-SWQ, the items related to romantic relationships and emotions dimension primarily focus on the quality of interactions with a romantic partner, while the items related to interacting with the opposite sex dimension in the CS-IRCDS primarily assess interactions with individuals of the opposite sex.

**Table 3:** Mean, standard deviation, and *t*-test in different dimensions of CS-IRCDS.

	2016	2023	*t*	*p*	*Cohen’s d*
Conversation and behaviors	4.563 ± 1.780	4.691 ± 2.014	−1.424	0.154	1.910
Interpersonal communication	4.197 ± 1.704	4.043 ± 2.160	1.665	0.096	1.964
Interacting with others	5.084 ± 1.455	5.539 ± 1.638	−6.191^**^	<0.001	1.556
Interacting with the opposite sex	5.242 ± 1.661	5.015 ± 1.839	2.728^**^	0.006	1.759

^**^p < 0.05.

According to Table 4, the mean and standard deviation of demographic variables among college students with romantic experience in the years 2016 and 2023 are presented. The mean and standard deviation of scores were compared between different gender subjects in 2 years, respectively. Independent sample *T*-test results showed that males in the CS-SWQ (*t* = 17.613, *p* < 0.001, *Cohen’s d* = 1.311) and the CS-LFQ (*t* = 3.015, *p* = 0.003, *Cohen’s d* = 0.212) had significant differences in 2 years, but no significant differences in the CS-IRCDS; females in the CS-SWQ (*t* = 9.145, *p* < 0.001, *Cohen’s d* = 0.719) and the CS-LFQ (*t* = 9.909, *p* < 0.001, *Cohen’s d* = 0.634) had significant differences in years, but no significant differences in the CS-IRCDS. Cohen’s d value of the CS-IRCDS is small, which should be noted. In 2023, males and females all had the lower scores in the CS-SWQ and CS-LFQ, and higher scores in the CS-IRCDS. In 2023, the average score of males in the CS-SWQ was lower than that of female students, while the average scores in the CS-IRCDS and CS-LFQ were higher than that of females. In 2016, the average scores in three questionnaires of males scored higher than females. Thus, the conclusion is that the three variables of males and females all changed before and after the epidemic.

**Table 4:** Mean and standard deviation of three questionnaires for demographic variables in 2016 and 2023.

	Year	Gender		Grade				Region	
		Male	Female	Freshman	Sophomore	Junior	Senior	City	Country
LF	2016	76.21 ± 15.25	70.03 ± 13.15	71.93 ± 12.27	70.55 ± 12.14	80.33 ± 12.21	69.75 ± 13.01	73.77 ± 16.19	72.23 ± 12.08
	2023	72.75 ± 17.72	62.38 ± 12.95	66.15 ± 14.65	64.47 ± 15.35	65.95 ± 15.37	70.73 ± 17.75	67.17 ± 16.31	66.47 ± 15.73
IRCDS	2016	19.72 ± 5.14	18.51 ± 5.21	18.75 ± 4.82	18.74 ± 4.89	20.03 ± 5.02	19.03 ± 6.05	19.25 ± 5.35	18.93 ± 5.01
	2023	19.53 ± 6.76	19.11 ± 6.02	20.15 ± 5.87	19.52 ± 6.83	19.52 ± 5.74	18.03 ± 6.45	19.79 ± 6.11	18.82 ± 6.53
SW	2016	228.57 ± 38.75	213.62 ± 33.39	221.08 ± 33.86	209.01 ± 30.89	236.15 ± 46.08	215.84 ± 30.06	222.92 ± 39.19	218.59 ± 33.58
	2023	188.20 ± 26.21	196.76 ± 20.37	190.49 ± 21.07	190.72 ± 26.93	194.40 ± 21.21	197.10 ± 22.56	192.71 ± 22.35	193.48 ± 24.40

We compared the mean score and standard deviation in three questionnaires for college students of different grades in 2016 and 2023 respectively, which are shown in Table 4. Independent sample *T*-test results showed that in the CS-SWQ, the scores of freshmen (*t* = 12.600, *p* < 0.001, *Cohen’s d* = 1.144), sophomores (*t* = 6.246, *p* < 0.001, *Cohen’s d* = 0.729), juniors (*t* = 11.489, *p* < 0.001, *Cohen’s d* = 1.417), seniors (*t* = 7.353, *p* < 0.001, *Cohen’s d* = 0.789) all had obvious differences in 2016 and 2023; in the CS-IRCDS, significant differences (*t* = −2.914, *p* = 0.004, *Cohen’s d* = 0.276) was only observed in freshmen in 2016 and 2023; in the CS-LFQ, the scores of freshmen (*t* = 4.815, *p* < 0.001, *Cohen’s d* = 0.451), sophomores (*t* = 4.291, *p* < 0.001, *Cohen’s d* = 0.411), juniors (*t* = 8.517, *p* < 0.001, *Cohen’s d* = 0.879) had significant differences in 2016 and 2023. In the CS-SWQ and CS-LFQ, the juniors had the highest average score in 2016, and seniors had the highest score in 2023. In the CS-IRCDS, the juniors had the highest average score in 2016, and freshmen had the highest score in 2023. Thus, the conclusion is that the three variables among the undergraduates of different grades changed before and after the epidemic.

We compared the mean score and standard deviation in three questionnaires for college students of different regions in 2016 and 2023, which are shown in Table 4. The results of independent sample T-test indicated that there were significant differences among urban undergraduates in years in the CS-SWQ (*t* = 14.562, *p* < 0.001, *Cohen’s d* = 1.070) and CS-LFQ (*t* = 6.216, *p* < 0.001, *Cohen’s d* = 0.406), but no significant differences in the CS-IRCDS; subjects in rural areas had significant differences in years in the CS-SWQ (*t* = 12.131, *p* < 0.001, *Cohen’s d* = 0.971) and CS-LFQ (*t* = 6.101, *p* < 0.001, *Cohen’s d* = 0.416), but no significant differences in the CS-IRCDS. The subjective well-being and love forgiveness among undergraduates from city and country all became lower in 2023. In the CS-SWQ, the mean score of city undergraduates was higher than country undergraduates in 2016, but lower than country undergraduates in 2023. Thus, the conclusion is that the three variables among the undergraduates of different regions changed before and after the epidemic.

The data collected in 2023 were analyzed. We compared the differences in the three questionnaires among college students with and without romantic experience, as shown in Table 1. Multivariate analysis of variance was employed to make a comparison between the scores among undergraduates with and without romantic experience in the questionnaires. The results showed that college students with and without romantic experience had significant differences in the CS-SWQ [*F* (1,1,480) = 17.335, *p* < 0.001, *η*^2^ = 0.012], CS-IRCDS [*F*(1,1,480) = 12.689, *p* < 0.001, *η*^2^ = 0.009] and CS-LFQ [*F*(1,1,480) = 31.005, *p* < 0.001, *η*^2^ = 0.021]. College students with romantic experience had higher average scores than those without romantic experience in the CS-SWQ and CS-IRCDS, and had lower average scores than those without romantic experience in the CS-LFQ. Hence, we conducted an independent sample *t*-test for the four dimensions in the CS-LFQ between college students with and without romantic experience. The results are shown in Table 5. College students with and without romantic experience had significant differences in the four dimensions. Students with romantic experience scored the highest in forgiveness dimension, while those without romantic experience scored the highest in revenge dimension. Simultaneously, the average score in avoidance dimension was higher among undergraduates with romantic experience than among those without romantic experience. However, in the other three dimensions, the average score was lower among undergraduates with romantic experience than among those without romantic experience. Thus, we conclude that college students with and without romantic experience had significant differences in love forgiveness.

**Table 5:** The difference of students with and without romantic experience in four dimensions of love forgiveness in 2023.

	Romantic experience	*N*	*Mean*	*SD*	*t*	*p*	*Cohen’s d*
Revenge	With	976	2.617	1.239	−27.082^**^	0.000	1.408
	Without	505	4.476	1.279			
Avoidance	With	976	3.757	1.551	2.572^*^	0.010	0.134
	Without	505	3.532	1.693			
Forgiveness	With	976	4.051	1.332	−4.405^**^	0.000	0.229
	Without	505	4.373	1.331			
Negative meditation	With	976	3.629	1.370	−2.498^*^	0.013	0.130
	Without	505	3.821	1.460			

^*^p < 0.05, ^**^p < 0.01.

### Relationship research

#### Correlation analysis

Three questionnaires total scores were transformed into Z-scores and then analyzed for correlation using SPSS. The results showing in Table 6 indicated significant correlations between each pair of the three variables.

**Table 6:** Correlation analysis.

		Love forgiveness	Interpersonal relationship	Subjective well-being
2016	Love forgiveness	1		
	Interpersonal relationships	0.365^**^	1	
	Subjective well-being	0.570^**^	0.570^**^	1
2023	Love forgiveness	1		
	Interpersonal relationships	0.257^**^	1	
	Subjective well-being	−0.063^**^	−0.305^**^	1

^**^p < 0.01.

In 2016, there was a weak correlation between the CS-LFQ and the CS-IRCDS (*r* = 0.365); the correlation between the CS-LFQ and CS-SWQ (*r* = 0.570) was moderate, as were the CS-IRCDS and CS-SWQ (*r* = 0.570). In 2023, there was a weak correlation between the CS-LFQ and CS-IRCDS (*r* = 0.0.257); the correlation between the CS-IRCDS and CS-SWQ (*r* = −0.305) was weak and negative, as were CS-LFQ and CS-SWQ (*r* = −0.063), which need careful attention. After the pandemic, the relationship between the three variables has changed.

#### Intermediate inspection

**Figure 2:**
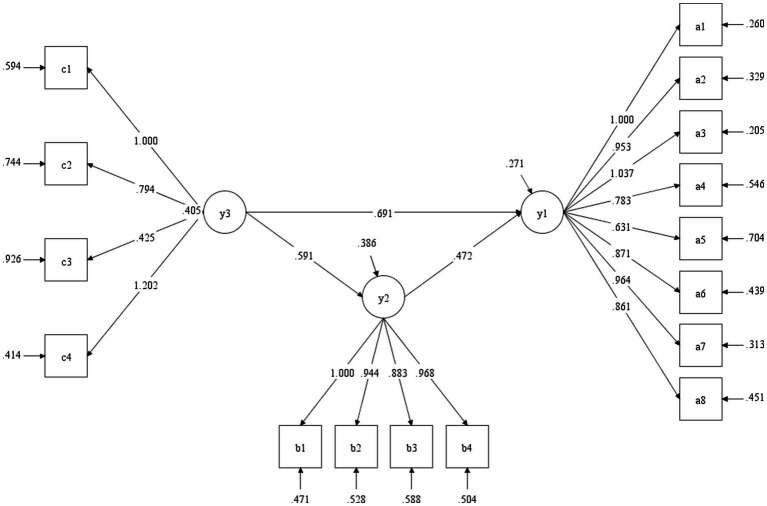
Mediating effect test in 2016.

In 2016, we employed Mplus 8.3 to evaluate the fitness level of a mediating effect model, which consisted of college students’ love forgiveness, interpersonal relationships, and subjective well-being (for a review, see Cheng et al., 2021). Table 7 shows the fitting index result of the mediation model (Model 1). The results showed interpersonal relationships significantly mediated the relationship between subjective well-being and love forgiveness among undergraduates. The mediation effect explained 40% of the total relationship, as shown in Figure 2.

**Table 7:** The fitting index result of the mediation mode.

	Model 1	Model 2	Model 3	Model 4
X^2^	1170.676	3038.453	2921.727	2816.099
*df*	101	101	126	126
*p*	0.000	0.000	0.000	0.000
RMESA	0.113	0.173	0.213	0.209
SRMR	0.060	0.133	0.180	0.162
CFI	0.852	0.632	0.054	0.537
TLI	0.824	0.563	0.481	0.515

**Figure 3:**
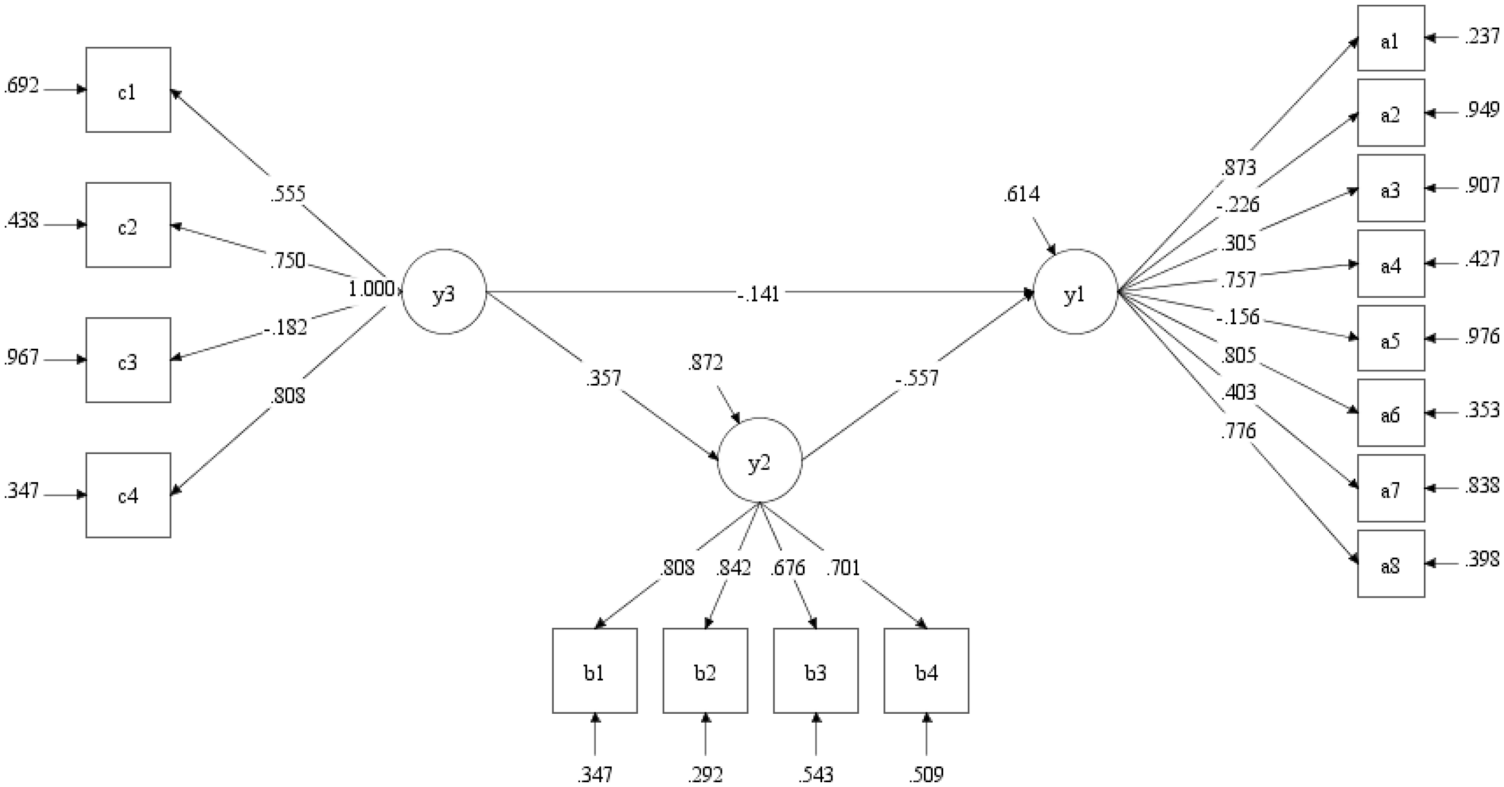
Mediating effect test in 2023.

In order to compare with the mediation effect model in 2016, we used the data from 2023 to evaluate the fitness level of the mediation effect model again. According to the mediating effect test procedure introduced by Wen et al. (2004), we first tested the direct effect between love forgiveness and subjective well-being, and the effect was significant (*t* = −10.871, *p* < 0.001). In the next step, we added interpersonal relationship and test the mediation model. The fitting index result (Model 2) was shown in Table 7. However, the CFI and TLI values were below 0.9 and they were even lower than that of 2016. Additionally, the SRMR value exceeded 0.08. These data suggested that the fitting index was not ideal and the model setting had problems. It can be found that the model established in 2016 no longer applies to 2023. Figure 3 shows the pathway of the mediation effect.

#### Moderator inspection

Since the three variables mediation model is no longer applicable to the post-pandemic period, we tried to examine the relationship between love forgiveness and subjective well-being by using demographic variables as the moderating variables. However, the operation results of Mplus8.3 showed that in the model with gender (Model 3) and region (Model 4) as dichotomous moderating variables, the difference test were all not significant (*p* > 0.05), and the model data are shown in Table 7. We then also tested the model with grade as a moderating variable, and the difference test between any two grades was not significant.

## Discussion

We investigated subjective well-being, interpersonal relationships, love forgiveness and the demographic variables of Chinese university students in 2016 and 2023, and compared the differences between the 2 years. We also compared the differences among college students with and without romantic experience in 2023. We found that before and after the COVID-19 pandemic, there was significant differences between three variables above among undergraduates, and there was also significant differences between the three variables of college students with and without romantic experience.

### Theoretical implications

We discovered notable differences in Chinese university students’ love forgiveness, interpersonal relationships, and subjective well-being between 2016 and 2023, the first hypothesis was valid. At first, compared with the period before the epidemic, undergraduates’ subjective well-being decreased significantly after the epidemic, which corresponds with the findings in Baetens et al. (2022) and Chen and Ye (2023). Krautter et al. (2022) found that during the epidemic, the subjective well-being of college students obviously decreased. In our opinion, the decline in college students’ subjective well-being can be attributed to two primary reasons. On the one hand, the epidemic has adverse effects on individuals’ psychological well-being. Many studies have shown that COVID-19 has affected people’s psychology to varying degrees (Bhattacharjee and Acharya, 2020; Chen et al., 2021). Studies from Japan highlighted the negative effect of COVID-19 on happiness (Shigemura et al., 2020). On the other hand, to minimize the likelihood of contracting the virus, during the epidemic, Chinese universities generally adopt closed-off management, that was, prohibited students from entering and leaving the school at will. Chinese universities also used the new forms of teaching that combining online and offline during the epidemic. However, the restriction of social distance (Esteves et al., 2021), activity limited to classroom and dormitory, and the new teaching form for a long time different from the tradition one brought by closed-off management may all have certain influence on students’ psychological status (Si et al., 2020; Chang et al., 2021; Xu et al., 2021). At second, in contrast to the period before the epidemic, the whole level of college students’ interpersonal relationships was slightly higher after the epidemic. The related researches showed that, the epidemic has changed people’s interpersonal relationships (Tribowo et al., 2021), and multiply affected the interpersonal relationships in school (Herrmann et al., 2021). However, the difference between college students’ interpersonal relationships became larger after the epidemic. This indicates that although college students’ interpersonal relationship has little change after the epidemic, some college students’ interpersonal relationship problems may be more serious. Why did the overall level of interpersonal relationship among Chinese college students not decrease but increase after the epidemic? Wang et al. (2021) pointed out that in Chinese collectivist culture-oriented environment, the fear and anxiety caused by the epidemic can trigger people’s proactive response (e.g., seeking social support). According to the research conducted by Tang et al. (2022), individual interpersonal relationship is positively correlated with social support. People seek mutual social support to maintain their interpersonal relationship and thus enhance it. At third, the love forgiveness among university students in the post-pandemic era has decreased, which is different from the findings of Cheng et al. (2021) before the epidemic. The epidemic has affected the intimate relationships (Tribowo et al., 2021), and increased the vulnerability of partnerships (Overall et al., 2021). Salo et al. (2022) found that stress during the COVID-19 pandemic had a detrimental effect on romantic relationships and negatively affects relationship functioning. It can be seen that the epidemic has a certain impact on individual romantic relationships. In addition, Zhang (2020) pointed out that the novel coronavirus pandemic, a major public health emergency, had an impact on Chinese college students’ concepts of love and marriage. During the epidemic, Chinese special epidemic prevention policies have led to an increase in the demand for essential materials among college students. At the same time, the conflict between husband and wife during the epidemic has deepened college students’ thinking about relationship maintenance. The changing views of love and marriage among undergraduates, coupled with a decline in the quality of the romantic relationships, could result in a lower level of love forgiveness.

We found that after the pandemic, undergraduates’ negative emotions significantly increased. This result is consistent with previous research findings (Cooper et al., 2021; Gao et al., 2022). In addition, the epidemic and prevention policies have led to a deterioration in undergraduates’ evaluation of themselves, their study, life and employment, and their feelings of getting along with others. The study by Krautter et al. (2022) showed that during the lockdown period, undergraduates’ satisfaction with life decreased. This indicates that the impact of the pandemic on individuals involves their learning, living, and work (Genc and Arslan, 2021). Besides, the behavior of undergraduates has been affected after the pandemic. Although the overall level of interpersonal interactions has improved, undergraduates reported a decline in their interactions with the opposite sex. This indicates that the impact of the pandemic on interpersonal interactions in schools is multifaceted (Herrmann et al., 2021). Further discussion is needed in the future regarding the effects on different interpersonal relationships in school.

We found that subjective well-being, interpersonal relationships, and loving forgiveness were positively correlated in 2016. However, love forgiveness was negatively correlated with subjective well-being, as were interpersonal relationships and subjective well-being in 2023. To be specific, after experiencing the pandemic, when undergraduates’ love forgiveness and interpersonal relationship become higher, their subjective well-being become lower. This indicates that the effects of the epidemic and the measures implemented to prevent and control it on Chinese undergraduates are significant. During the epidemic, although college students still maintain interpersonal or romantic relationships, the recurrence and uncertainty of the epidemic has increased their feelings of uncertainty, insecurity, anxiety, panic and other emotions. In addition, Chinese special epidemic prevention policies in universities broke students’ life rules and seriously affected their psychological health (Wu, 2023). All these resulted in a decrease in the subjective well-being among undergraduates.

We found that the subjective well-being and love forgiveness among university students of different genders were significantly different in 2016 and 2023, and hypothesis 2. was confirmed. At first, after the epidemic, undergraduates’ the subjective well-being was still above the average level, and female students’ subjective well-being was higher than male students. This finding conforms to the research of Zhang F. et al. (2021). Undergraduates exhibit favorable psychological traits, possess high resilience toward stress, and can readily acclimate to new surroundings (Xue et al., 2022). The study has shown that on the whole, Chinese undergraduates have a high level of mental toughness (Feng et al., 2016), which can effectively predict well-being (Wang and Wang, 2013). Meanwhile, female mental toughness was better than male (Li et al., 2022), so females’ subjective well-being was higher than males. At second, before and after the epidemic, interpersonal relationships for male students have consistently been better than female students, which is consist with the results of Gao (2022) about Chinese middle school students. The study by Guo et al. (2022) revealed that male university students had better interpersonal relationships in dormitories compared to female students. The interpersonal relationships of male college students have worsened, while those of female college students have improved. This suggests that the pandemic may have had a positive impact on female university students’ interpersonal relationships and a negative impact on female university students’ interpersonal relationships. We consider that this may be due to the fact that, in the face of the pandemic, female tended to get help through interpersonal interaction and were more likely to receive help and support from others (Zou et al., 2021), thus having better interpersonal relationships. At third, after the epidemic, male students exhibited greater love forgiveness than female students, which is consistent with the results before the epidemic (Yu, 2019; Cheng et al., 2021). Due to the positive correlation between love satisfaction and love forgiveness (Liu, 2017) and male college students exhibited higher love satisfaction levels compared to female college students (Yu, 2019), so males’ love forgiveness was higher than females.

We found that before and after the epidemic, students in four grades had noticeable differences in their levels of subjective well-being; freshmen showed obvious differences in their levels of interpersonal relationships; freshmen, sophomores and juniors had significant differences in love forgiveness. Hypothesis 3 was partly confirmed. At first, compared with the period before the epidemic, the subjective well-being of students in the four grades decreased after the epidemic. It can be seen that the epidemic has brought different detrimental effects on the subjective well-being among university students in the four grades. At second, after the epidemic, the interpersonal relationship of juniors and seniors decreased, while that of freshmen and sophomores increased. Among them, the lower grade students had the best interpersonal relationship, and the higher grade students had the worst interpersonal relationship. This is completely contrary to the results of the studies before the epidemic (Wang, 2015; Cheng et al., 2021), but is similar to the result of Zhang et al. (2022). We believe that the change of interpersonal relationship between different grades is mainly related to epidemic prevention policies. The study has shown that college students’ interpersonal relationship is related to social support (Elliott and Gramling, 1990; Zhang X. et al., 2021). During the epidemic, according to Chinese epidemic prevention policy, universities generally adopted closed-off management, and college students received social support mainly from classmates and teachers. In middle schools, students can study at home and receive social support from peers, teachers and family members. Compared with lower grade students, higher grade students experienced longer close-off in college, had less social support, and hence may have more interpersonal problems. At third, after the epidemic, only the love forgiveness of seniors increased slightly, while that of the other three grades all decreased. Among them, seniors had the highest level of love forgiveness. This is inconsistent with the results before the pandemic (Li et al., 2010; Xu and Liang, 2013). Shen and Liu (2022) pointed out that at present, the difficulties young people encounter in romantic relationship can be summarized in two aspects: on the one hand, romantic relationship is not the most urgent pursuit of some college students, and they show an attitude of “everything goes with the wind” when facing their feelings; on the other hand, some college students are reluctant to pursue love due to personality and economic conditions, and their attitude toward love is “prefer not to love, rather than hurt.” Affected by the epidemic, freshmen, sophomores and juniors have become more serious about romantic relationship. They are also more reluctant to forgive when faced with hurt in a relationship. As seniors are about to graduate, the problem of employment or admission is more troubling to them, and their attitudes toward romantic relationship become more spontaneous. Seniors may pay less attention to the hurt in romantic relationships than students in other grades, and thus are more likely to forgive. The differences of undergraduates’ love forgiveness need to be discussed more in future studies.

We found that before and after the epidemic, college students in different regions had obvious differences only in love forgiveness and subjective well-being. Hypothesis 4 were partly confirmed. After the epidemic, the subjective well-being and love forgiveness among undergraduates from city and country decreased. But rural college students’ subjective well-being was higher than urban college students, which is different from the situation before the epidemic (Liu and Wang, 2012; Cheng et al., 2021). The study conducted by Guo et al. (2022) after the pandemic found no significant differences in subjective well-being among undergraduates in different regions. The epidemic did affect college students in different degrees and seemed to affect the students from the urban areas more. There are two reasons for this phenomenon: from one perspective, population in urban areas is more dense, and the risk of virus infection is greater. Students in city have experienced more anxiety during the epidemic, thus had lower subjective well-being; from another perspective, the management of Chinese rural areas were a weak spot for epidemic prevention due to the wide geographical area and poor economic foundation. Compared with urban areas, epidemic prevention policies in rural areas were relatively relaxed, and college students in rural areas were less affected. Therefore, rural college students’ subjective well-being was higher than urban college students.

We found that college students without romantic experience and those with romantic experience had significant differences in three variables. Hypothesis 5 was confirmed. First of all, compared with the students with romantic experience, the students without romantic experience had lower subjective well-being lower interpersonal relationship, and higher love forgiveness. Undergraduates who have romantic experience had higher level of subjective well-being, which is consistent with the findings in existing researches on marriage. To provide an instance, studies have pointed out marriage can significantly improve individual subjective well-being (Lee and Ono, 2012; Hu et al., 2022). This result is similar to the findings of Guo et al. (2022). They found that college students in satisfying romantic relationships had higher levels of subjective well-being. Again, university students with romantic experience had higher interpersonal relationship, which is consist with the result of Luo et al. (2013). They found that military personnel in romantic relationships experienced less interpersonal distress compared to those who were not in relationships. However, Xiong and Liang (2020) pointed out that romantic relationships may lead to increased interpersonal distress among undergraduates. The more individuals value love and have higher expectations, the more likely they are to experience interpersonal distress (Luo et al., 2013). What is more, the love forgiveness of college students without romantic experience was higher than those with romantic experience. The love forgiveness of college students without romantic experience was their imagined forgiveness when facing the hurt in romantic relationship. This suggests that in actual romantic relationship, college students show a lower level of love forgiveness than they think. We think that this gap between imagination and reality may be affected by a variety of factors, such as love satisfaction, love psychological maturity and so on. More research is needed to discuss this. Last but not the least, we also found that in the four dimensions of the CS-LFQ, there are significant differences between college students with and without romantic experience. College students with romantic experience scored the highest on forgiveness and the lowest on revenge, while college students without romantic experience scored the highest on revenge and the lowest on negative meditation. Zhang and Fu (2013) found that Chinese college students with romantic experience are more inclined to meditate negatively than to retaliate. In other words, in imaginary relationships, college students are more likely to take revenge on other people and least willing to meditate negatively; in actual relationships, college students are more likely to forgive and least willing to revenge. The performance of college students in imagined romantic relationships may represent their implicit level of love forgiveness, while the performance of those with romantic experience show their explicit level of love forgiveness. The study has shown that implicit interpersonal forgiveness level and explicit interpersonal forgiveness level are two different constructs, and there is no correlation between them (Wen and Chen, 2022). Therefore, the differences in the dimensions of love forgiveness of undergraduates with and without romantic experience can be understood, but the reasons for the differences still need to be more discussed in the future.

### Practical implications

Firstly, we found that the epidemic and prevention measures had an impact on the mental health and behavior of Chinese university students. This suggests that the Mental Health Departments should take the mental state and problems of college students into account, and take the physical and mental health of university students as indicators that must be considered in the formulation of epidemic prevention policies in universities in the future. The change in the indicators among university students also suggests that the Chinese government needs to attach importance to the physical and mental health of different groups in the post-epidemic era, and make timely intervention measures to maintain people’s health.

Secondly, the decrease in subjective well-being and increase in interpersonal issues among university students suggest that universities need to give priority to students’ mental health. They should provide more psychological counseling services and health education to guide students in adapting to campus life more effectively. Considering that there are differences in well-being and interpersonal relationships among students of different grades, regions, and genders, the universities must develop different strategies for providing counseling and education to different types of students.

Thirdly, college counselors need to change their previous work mindset when assisting students. Due to the changing relationship between subjective well-being and interpersonal relationships among university students, those with good interpersonal relationships may have lower subjective well-being. Therefore, counselors should not overlook those students with good interpersonal relationships when identifying those with lower subjective well-being. College students’ forgiveness level in romantic relationships has decreased, indicating a shift in their beliefs and a greater likelihood of conflicts and issues arising. Counselors must strengthen their focus on college students in romantic relationships. At the same time, college students who lack romantic relationship experience tend to have lower subjective well-being and weaker interpersonal relationships, so counselors need to provide them with more social support.

Lastly, after experiencing the pandemic, undergraduates are more likely to choose forgiveness and avoidance rather than negative meditation when faced with hurts in romantic relationships. This suggests that they are changing their coping strategies in intimate relationships. Male university students tend to have higher levels of forgiveness in romantic relationships compared to females. When dealing with issues related to students’ intimate relationships, counselors need to consider their gender and develop counseling plans that encourage students to confront the hurts in their romantic relationships and guide them in better managing their love relationships.

### Advantages and limitations

The advantages of this research are as follows: (1) By comparing the data before and after the epidemic, it found that the COVID-19 epidemic and Chinese special epidemic prevention policy had certain effects on university students’ psychological and behavioral aspects, which provides evidence for the related researches on the effects of the epidemic. This can arouse social attention to the physical and mental health conditions of undergraduates, and also serve as a reminder for relevant departments to consider people’s various indicators when formulating epidemic prevention measures in the future. (2) This study found differences in indicators among undergraduates in different grades, regions, and genders, which is beneficial for universities and other researchers to understand the basic conditions of different types of students after the epidemic. (3) This study found changes in the relationship between subjective well-being, interpersonal relationships, and love forgiveness among undergraduates, suggesting that other researchers should introduce new variables to examine the relationship between them. (4) This research also found college students with and without romantic experience had certain differences in subjective well-being and interpersonal relationship, and students’ imagined level of love forgiveness is higher than their actual love forgiveness. This provides new ideas for other researchers in the following study.

Nevertheless, this research has certain limitations that need to be acknowledged: (1) The subjects of this study before and after the epidemic were not the same group of college students, and the survey was conducted over a period of 7 years. There must be some errors in the comparison results, which should be paid attention to. (2) All questionnaires distributed in 2023 were online, which is different from 2016. Research differences in how tools were distributed may affect the accuracy of the research. (3) The pandemic has changed people’s lives. The functioning of young people has changed significantly over the past 7 years, and it is possible that this transition to online functioning has not affected changes in young people as much as it has in older people. This study was limited to college students, which cannot fully explain the impact of the epidemic on people. More researches on other age groups are needed in the future. (4) The subjects in 2016 and 2023 were mainly from economically developed regions of China. The representativeness of the sample cannot be guaranteed and the external validity of the research results has yet to be verified. (5) After the epidemic, the relationship between love forgiveness, subjective well-being, and interpersonal relationship among undergraduates has changed. Due to time constraints, this study did not explore other variables affecting the above relationship.

### Directions for future research

First of all, interpersonal relationships no longer mediate between subjective well-being and love forgiveness among undergraduates. To explore the relationship between these three variables, it is necessary to consider the inclusion of other variables, such as relationship satisfaction, social support, psychological resilience and so on. Future research can delve deeper into the discussion. Secondly, Chinese special epidemic prevention policies indeed exhibited certain effects on university students’ psychology and behavior. Future research can explore the impact of the pandemic on other age groups. Additionally, comparing the psychological and behavioral differences among college students in different countries can demonstrate the effects of different preventive policies. And then, this study did not specifically distinguish the group of college students with romantic experience. Future research can examine the level of love forgiveness among undergraduates with different numbers of romantic experiences and varying levels of relationship satisfaction. Finally, the difference between undergraduates’ imagined and actual level of love forgiveness is worth further discussion by researchers. What factors contribute to the variation in levels of love forgiveness? Is this variation present within the same individual? Future research can adopt a longitudinal approach to delve into the mechanisms of changes in love forgiveness.

## Data availability statement

The original contributions presented in the study are included in the article/supplementary material, further inquiries can be directed to the corresponding author.

## Ethics statement

The studies involving human participants were reviewed and approved by Ethics Committee of Huaiyin Normal University. The patients/participants provided their written informed consent to participate in this study.

## Author contributions

TC was responsible for contacting the subjects, handing out questionnaires, processing data, and writing the article. LQ participated in the research design, provided specific ideas for the paper writing, and made constructive comments on the data analysis process. HF put forward many constructive suggestions for the revision of the article. All authors contributed to the article and approved the submitted version.

## Conflict of interest

The authors declare that the research was conducted in the absence of any commercial or financial relationships that could be construed as a potential conflict of interest.

## Publisher’s note

All claims expressed in this article are solely those of the authors and do not necessarily represent those of their affiliated organizations, or those of the publisher, the editors and the reviewers. Any product that may be evaluated in this article, or claim that may be made by its manufacturer, is not guaranteed or endorsed by the publisher.
